# Time-coursed transcriptome analysis identifies key expressional regulation in growth cessation and dormancy induced by short days in *Paulownia*

**DOI:** 10.1038/s41598-019-53283-2

**Published:** 2019-11-12

**Authors:** Jiayuan Wang, Hongyan Wang, Tao Deng, Zhen Liu, Xuewen Wang

**Affiliations:** 1 College of Forestry, Henan Agricultural University, Zhengzhou, Henan 450002 China; 2 School of life science, Liaoning University, Shenyang, 110000 China; 3 Key Laboratory for Plant Diversity and Biogeography of East Asia, Kunming Institute of Botany, Chinese Academy of Sciences, Kunming, 650201 China; 4 Department of Genetics, University of Georgia, Athens, 30602 USA

**Keywords:** Gene regulation, Light responses, Plant hormones

## Abstract

Maintaining the viability of the apical shoot is critical for continued vertical growth in plants. Terminal shoot of tree species *Paulownia* cannot regrow in subsequent years. The short day (SD) treatment leads to apical growth cessation and dormancy. To understand the molecular basis of this, we further conducted global RNA-Seq based transcriptomic analysis in apical shoots to check regulation of gene expression. We obtained ~219 million paired-end 125-bp Illumina reads from five time-courses and *de novo* assembled them to yield 49,054 unigenes. Compared with the untreated control, we identified 1540 differentially expressed genes (DEGs) which were found to involve in 116 metabolic pathways. Expression of 87% of DEGs exhibited switch-on or switch-off pattern, indicating key roles in growth cessation. Most DEGs were enriched in the biological process of gene ontology categories and at later treatment stages. The pathways of auxin and circadian network were most affected and the expression of associated DEGs was characterised. During SD induction, auxin genes *IAA*, *ARF* and *SAURs* were down-regulated and circadian genes including *PIF3* and *PRR5* were up-regulated. *PEPC* in photosynthesis was constitutively upregulated, suggesting a still high CO_2_ concentrating activity; however, the converting CO_2_ to G3P in the Calvin cycle is low, supported by reduced expression of *GAPDH* encoding the catalysing enzyme for this step. This indicates a de-coupling point in the carbon fixation. The results help elucidate the molecular mechanisms for SD inducing dormancy and cessation in apical shoots.

## Introduction

Plant dormancy is a temporary, visible suspension in meristem, which is an adaptation for survival and growth in response to environmental stresses, especially for temperate plants. Most perennial plants undergo seasonal cycling of dormancy and regrowth, e.g., a typical dormancy in fall or winter and regrowth the next spring. Dormancy and its release affect the annual vertical growth of perennial plants. Dormancy studies have been carried on some plants, including model plants *Arabidopsis* and *Populus*^1^ and perennial grasses^2^.

The short day (SD) photoperiod is known to play a crucial role in plant growth cessation, dormancy induction and dormant bud formation^1^. *PHYTOCHROME* (*PHY*) encodes a photoreceptor for day length signal perception that passes the SD signal to an internal regulatory network via PHY-interacting transcription factors (PIFs). The expression of *PIF4* and *PIF3* is up-regulated under SD in *Populus*^3^. In trees, the seasonal cycling between growth and dormancy is controlled by the *CONSTANS* (*CO*) and *FLOWERING LOCUS T* (*FT*) module^1^. Down-regulated *FT* expression under SD leads to growth cessation and dormancy in *Populus*. The *FT* expression is positively regulated by *CO*, which is very low in the daytime under SD. The phytochrome responses to day length is mediated through *CO*^1^. Over-expression of the basic leucine zipper transcription factor *PtFD1*, which interacts with *FT*, abolishes growth cessation and bud formation induced by SD in *Populus*^4^. Low expression levels of *PHYA* and *CENTRORADIALIS-LIKE 1/TERMINAL FLOWER 1* (*CENL1/TFL1*) in rib meristem induce terminal bud formation and subsequent dormancy in *Populus* under SD^5^. The signal for dormancy originates from the rib meristem and shoot apical meristem, instead of the leaf^5^. A microarray-based gene expression study in grapevine (*Prunus persica*) showed that expression of *C3HC4-TYPE RING FINGER* and *NAC DOMAIN CONTAINING PROTEIN 19* is significantly altered during SD-induced dormancy^6^.

Plant hormones play a critical role in regulating dormancy in woody plants^7,8^. Abscisic acid (ABA) is the main hormone that responds to various environmental stresses, associates with photoperiodic pathways and affects the circadian clock, therefore leading to dormancy^3,7^. Over expression of *ABSCISIC ACID-INSENSITIVE3* (*ABI3*), which encodes an ABA-related transcription factor, can induce dormancy in *Populus*^3^. The ABA concentration accumulates gradually within the first three weeks of SD treatment and peaks at day 21, indicating a potential role in the bud dormancy response^6^. Twelve ABA metabolism-related genes (9 *PpCYP707As* and 3 *PpNCEDs*) were identified in peach seeds and during bud dormancy. However, the central role of ABA in maintaining bud dormancy is still not well understood^8^. In addition to ABA, the gibberellins (GAs) are also important in controlling the processing of bud dormancy^3,7,9^. GAs can promote growth and various developmental processes, including stem elongation, germination, and release of dormancy^9^. Lower GA levels result in seed dormancy in *Arabidopsis*^8,10^, which is regulated by the REVEILLE1 transcription factor and phytochrome B^11^. Indole acetic acid (IAA) often shows the opposite accumulation patterns to ABA. The concentrations of IAA in the shoot apical meristem are lower during bud dormancy but increase from dormancy to activation^12,13^. Array-based gene expression analysis shows that genes in GA, ethylene, auxin and ABA signalling pathways are regulated in bud dormancy in grape^9^. Reduced levels of ethylene, GA, and auxin or increased levels of ABA are responsible for growth cessation^7,14^. Some crosstalk pathways exist in bud dormancy^7,8,12^. The balances between ABA and GA, ABA and ethylene are essential for dormancy regulation^7,15^.

Carbon fixation also plays role in the transition to dormancy. Carbon fixation is the bottleneck of photosynthesis for solar energy storage and carbon compound assimilation from inorganic carbon dioxide in the atmosphere. C3 and C4 carbon fixation pathways are the most common in land plants. C4 is a more efficient pathway because C4 has an additional C4-dicarboxylic acid cycle that separates the carbon gain in the night and day time^16,17^. Phosphoenolpyruvate carboxylase (PEPC) and ribulose-1,5-bisphosphate carboxylase oxygenase (Rubisco) are the two key enzymes for carbon fixation. At night, CO2 is fixed into malate by PEPC. In the day, Rubisco re-fixes the CO2 released from malate or directly from the atmosphere^16,17^. High PEPC activity is correlated with high malate accumulation, and the carbon fixed by Rubisco is preferentially used for export in leaf tissues^18^. Several studies have shown that carbon fixation is regulated under dormancy. A seasonal study demonstrated that the carbon flux was decreased in the deciduous tree trunk at the end of growth season^19^. Carbon turnover may be enhanced in the phloem during dormancy, as measured by basipetal isotope signal depletion^20^. *RUBISCO* expression is decreased during ABA-induced dormancy^21^.

Other factors may also affect dormancy. Low temperature impacts dormancy, and an extended time at low temperature is also required for dormancy release in some plants. *DORMANCY ASSOCIATED MADS-BOX (DAM*) transcription factors in peach are up-regulated during cool temperature-induced dormancy^22^ and repressed by epigenetic modification during dormancy release^23,24^, and a deletion in *DAM* prevents terminal bud formation^25^. Dormancy release is regulated by *FT* and *TFL1* during vernalisation, which is reviewed by Brunner *et al*.^26^. miRNAs also regulate dormancy release after chilling in *Populus*^27^.

*Paulownia*, also called princess tree, is a fast-growing tree species that belongs to the family *Paulowniaceae*. The genus *Paulownia*, native to China, is composed of nine species and a few natural hybrids distributed worldwide^28^. *Paulownia* has been grown for the wood industry^28,29^ and soil protection because of its well-developed root system^30^. In the next year, the terminal buds of *Paulownia* will die, and only axillary buds will grow, which results in a false dichotomous branching pattern. It is still unknown when the transition occurs between dormancy and death in *Paulownia* buds. However, the terminal bud of a few *Paulownia* genotypes in tropical places can regrow in the subsequent year^31^, indicating this is controlled by genetic variation. A short day length introduced growth cessation in *Paulownia*^32^. Several studies on *Paulownia*, reviewed by Wang *et al*.^33^, have mostly focused on morphological, physiological and horticultural investigations into *Paulownia* terminal buds. Little is known about the molecular mechanism of *Paulownia* dormancy.

In this study, we initiated and focused on gene discovery and gene regulation in the induction of dormancy or growth cessation by a short-day photoperiod in *Paulownia* in a green house. We used RNA-Seq technology and validated by RT-qPCR to perform genome-wide transcriptomic analysis in time courses from active growth to growth cessation in the shoot tips of *P*. *tomentosa*. We identified differentially expressed genes and their regulatory patterns during the induction of growth cessation and dormancy. We further investigated responsible genes in plant hormone pathways such as IAA, circadian regulation such as *PIF3*, carbon fixation metabolism and energy ATP production in photosynthetic processes. The results provide better understanding of the molecular mechanisms in SD-induced growth cessation and dormancy induction in *Paulownia* shoot tips.

## Results

### Short day induces growth cessation and dormancy in *Paulownia*

**Figure 1:**
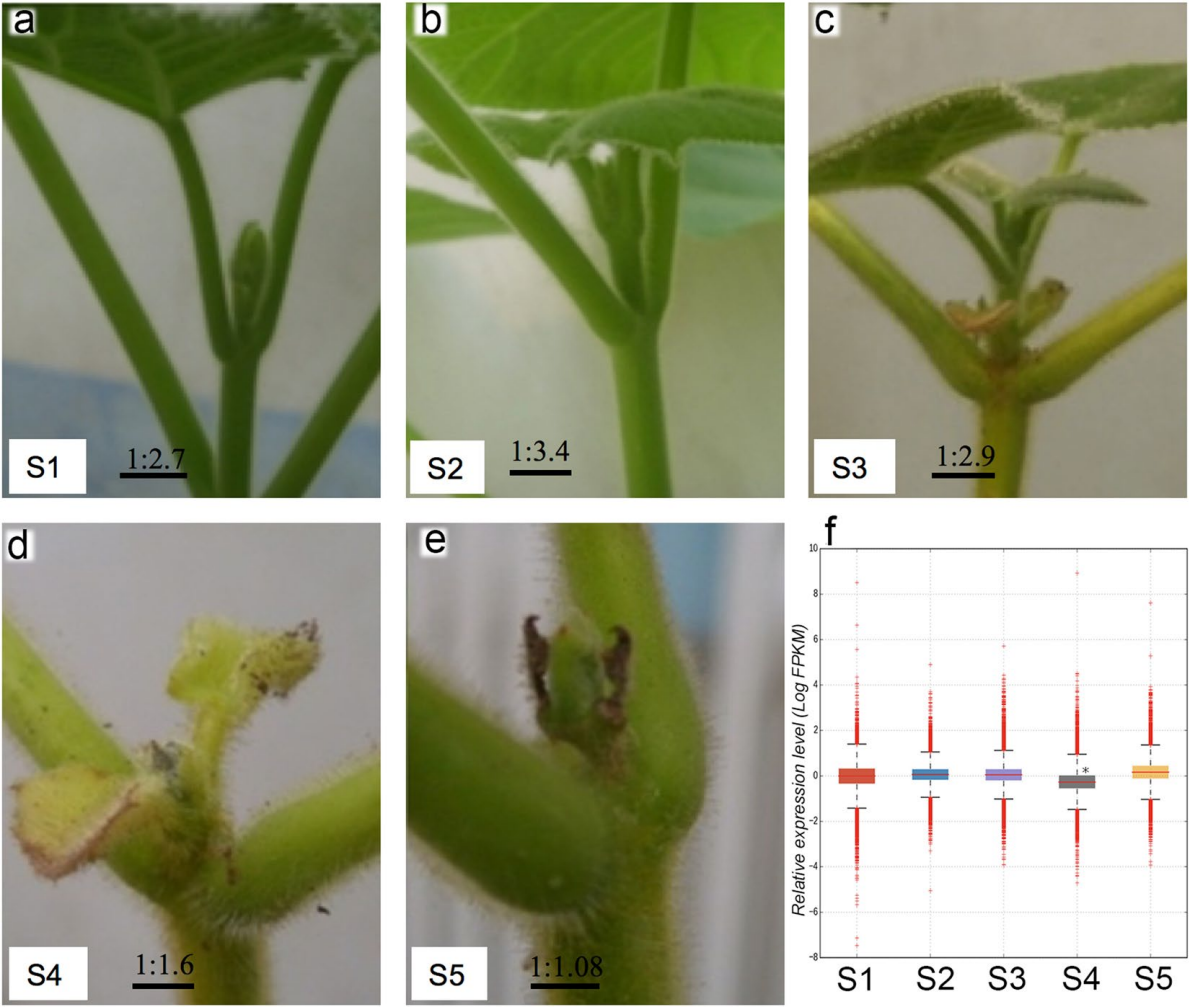
Phenotype of *Paulownia* shoot tips at stages before and after short-day treatment. Image a-e show the shoot tip of *Paulownia*, taken on at the start point on Aug 1st (S1) under long-day light cycles of 16 h light and 8 h darkness, and then at every five-day interval on Aug 6th (S2), 11th (S3), 16th (S4) and 21st (S5) under short-day light cycles of 8 h light and 16 h darkness. Image f shows the boxplot distribution of gene expression of all genes in shoot tips at each stage. *Represents significance p < 0.05 compared with any other stages. The gene expression value was presented after log2 (FPKM) transformation.

We treated one-year-old young trees propagated from roots of *Paulownia* cultivar *P*. *tomentosa* with short-day cycles of 8 h light and 16 h darkness in a greenhouse during the fastest growing season in August. 0.5 cm shoot tips from eight trees were sampled on Aug 1^st^ (S1) as a control before the SD treatment, and similar tissues were collected at five-day intervals on Aug 6 (S2), 11 (S3), 16 (S4) and 21 (S5). Obvious growth cessation and dormancy induction in shoot tips were observed after SD treatment (Fig. 1a–e), whereas the shoot tips under a long-day photoperiod were still in a fast-growing condition, the same as at S1. The apical shoots from stage S4 had similar phenotype to that of dormant shoots at the end of September when the growth was stopped and enlarged vessel cells and increased xylem were formed in the cambia zone of apical shoot^34^. We found that the resulting terminal buds did not regrow in the following year. However, after treating one-year-old trees with constant light, the trees exhibited a very late growth cessation followed by dormancy and terminal buds can regrow in the next year. The terminal buds of one-year-old trees did not re-grow in the coming year even after being transferred to a tropical area in Guangzhou, China (GPS location 23.157 N, 113.359 E). Taking these results together, we concluded that SD, instead of LD, induced dormancy and death in the terminal shoots of the *Paulownia*.

### *De novo* assembly of transcripts in *Paulownia*

Transcriptomes from each stage of the collected samples were examined with RNA-Seq technology. More than 39.7 million paired-end 125-bp Illumina reads were generated from mRNA extracted from samples at each stage, totalling 219,619,968 reads with a quality score Phred Q30 > 92% from the five stages (Supplementary Table S1). All reads were *de novo* assembled into transcripts with Trinity software (version 201308)^35^, and 49,054 unigenes were obtained. More than 75% of the paired reads were properly mapped back to the unigene assembly. The RNA-Seq reads have an average coverage depth of 220 X and unigenes have average length of 853 bp. The RNA-Seq data and unigene assemblies are publicly available at NCBI (Supplementary Table S1).

### Functional annotation of transcripts

The transcripts were annotated by similarity searching against the NR databases of NCBI, EggNOG, KEGG and InterPro (E-value ≤ 10–10 for Pfam, E-value ≤ 10–5 for others). A total of 54,880 (55%) transcripts were annotated with at least one hit from the databases (Supplementary Table S1). A total of 24,184 (24%) transcripts were functionally annotated in the orthologous database EggNOG. Among the orthologs, the largest portion of the transcripts (14,175; 43%) belonged to the class of cellular process and signalling, dominated by functional groups “posttranslational modification, protein turnover, chaperones,” “signal transduction mechanisms” and “intracellular trafficking, secretion, and vesicular transport” (Supplementary Table S2). A total of 9150 (28%) transcripts were in the class of information storage and processing, dominated by “transcription.” Of the transcripts, 9475 (29%) belonged to metabolism, dominated by “carbohydrate transport and metabolism” (Supplementary Table S2). Further enrichment analysis by using KOBAS^36^ revealed that transcripts in *Paulownia* were mapped to 255 KEGG pathways.

### Overview of differential gene expression induced by short day treatment

We measured the genome-wide transcript abundance from our RNA-Seq data in fragments per kilobase length per million reads (FPKM). The results showed that the average abundance of all transcripts display a decreased trend with increasing days of SD treatment, reaching a lower level after 15 days of treatment at stage S4 (*p < 0*.*05*, Fig. 1f). Pearson correlation analysis showed that the expression levels were more similar at two successive stages, indicating a gradual change of expression in apical shoots (Supplementary Fig. S1).

**Figure 2:**
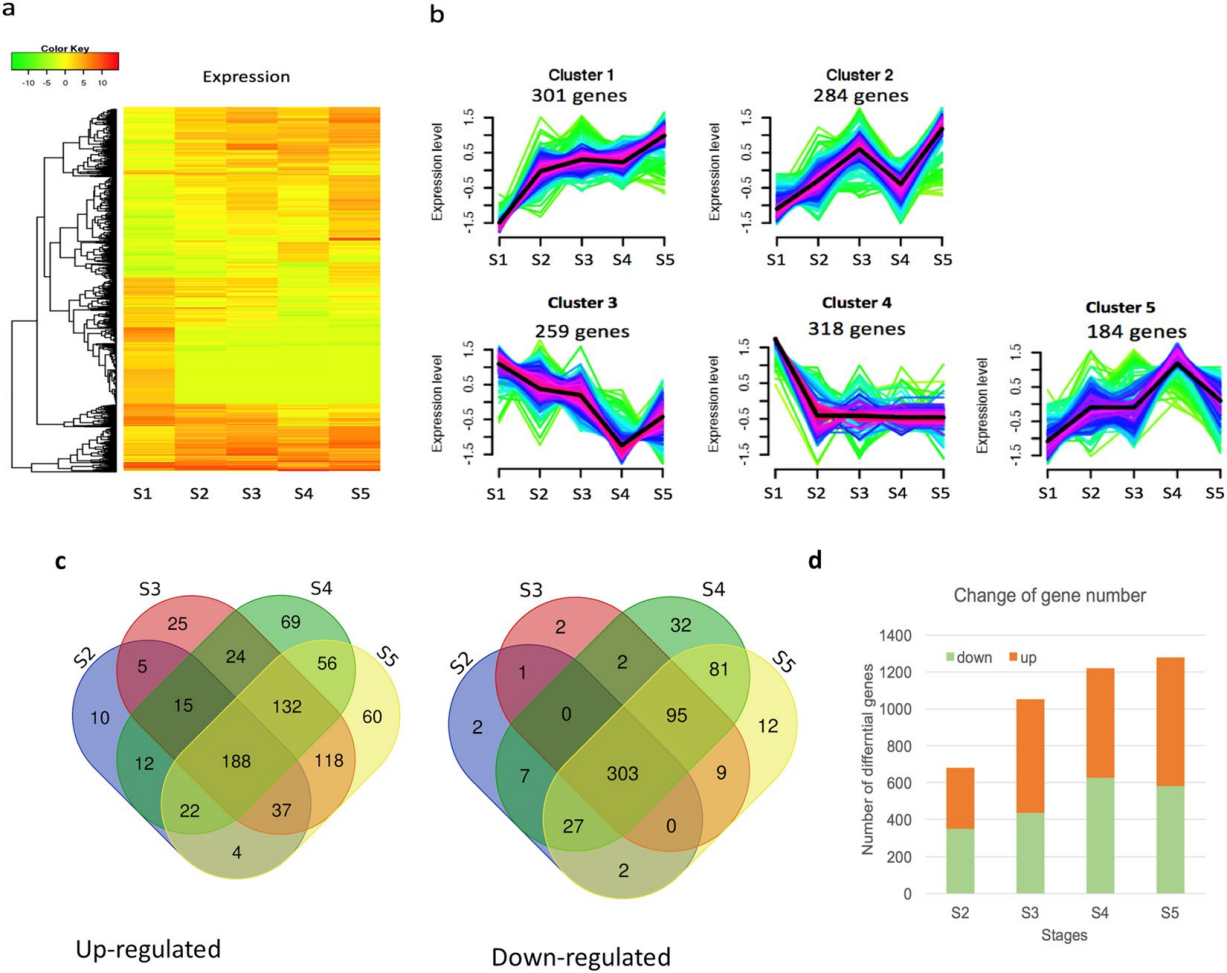
Expression patterns of differential genes in time-course series to short-day treatment in *Paulownia*. Genes expression displayed here was at least 2 folds of change and statistically p < 0.0001. S2, S3, S4, and S5 represent the shoot tip samples after 5, 10, 15, and 20 days of short-day treatment while S1 represents for long day condition before treatment. Heatmap (**a**) shows the gene expression level in color after log2 transformation of FPKM value. Each row represents expression level of one gene at different samples. The tree in the left shows the clusters of expression pattern. Image (**b**) shows the clustered patterns of gene expression. Venn diagram (**c**) shows the common and specific differentially up-regulated and down-regulated genes across samples. Image d shows the number of up- and down- regulated genes relative to S1.

We then investigated the differential expression (DE) of genes in response to the SD treatment in time courses and then focused on the DE compared with the starting point, as a control under LD (16 h light and 8 h darkness) just before the SD treatment. In total, the expression of 1540, 1540 and 1346 genes were changed at least two-fold (*p* < 0.0001), four-fold (*p* < 0.0001) and eight-fold (*p* < 0.0001), respectively, suggesting an expressional regulation of many genes in response to SD treatment (Supplementary Table S3). Most (87.4%) of the DE genes that had two or four-fold change also had an eight-fold change, suggesting that a short-day photoperiod induced a dramatic change in gene expression (Supplementary Table S3, Fig. 2a). Then, we focused on the 1540 DE genes for subsequent analyses. The expression patterns fell into five major clusters, and 318 genes in cluster 4 were constitutively switched off after SD treatment (Fig. 2a,b). Across all SD stages, 491common DE genes, consisting of 188 up-regulated and 303 down-regulated DE genes, were identified compared with LD stage S1 (Fig. 2c). Comparison revealed that more DE genes were differentially regulated with accumulated SD treatment (Fig. 2d). The information of DE gene IDs and their up- or down-regulation at each SD stage is available at Supplementary Table S4.

### Gene ontology (GO) classification and involved pathways of differentially expressed genes

**Figure 3:**
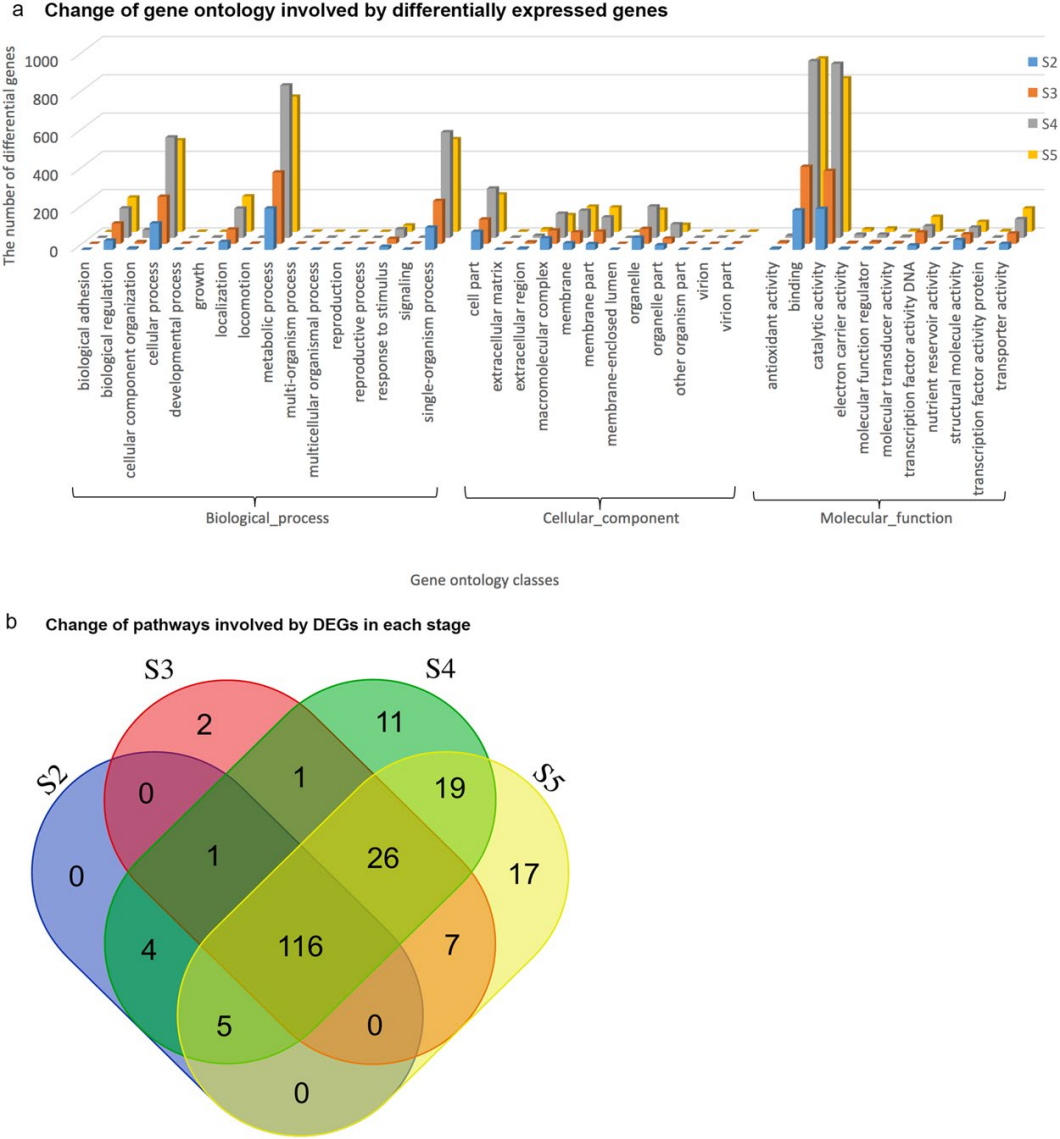
Chariterisation of GOs and enriched pathways of differentially expressed genes in shoot tips under short-day photoperiods. Image a shows enriched gene ontologys. Image b shows the number of pathways mapped by differentially expressed genes (DEGs) against database Kyoto Encyclopedia of Genes and Genomes. S2, S3, S4, and S5 represent the apical shoot tip samples or stages after 5, 10, 15, and 20 days of short-day treatment.

The annotation of DE genes was retrieved from unigene annotation for functional characterisation and annotation results against database NR, Interpro, EGGNOG, KEGG were available in Supplementary Tables S5–S8, respectively. In total, 860, 1414, 2983 and 2734 of the DE transcripts from stages S2, S3, S4 and S5, respectively, were assigned GO terms (Supplementary Table S6). Across all stages, the biological process was the most enriched GO category, followed by the molecular function category (Fig. 3a). Metabolic process, single-organism process, cellular process, localisation, and biological regulation were the most abundant groups in the biological process category. In the molecular function category, binding and catalytic activity were the most abundant groups. In the cellular component category, cell part, macromolecular complex, membrane, membrane part and organelle were the most abundant groups, though fewer genes were involved than in the other two categories (Fig. 3a).

To understand the regulatory networks of DE genes, we conducted pathway mapping against a pathway database KEGG using the KOBAS tool (version 3.0). The results revealed that DE genes were mapped to 209 pathways. Of those, 116 pathways were common in all stages of SD treatment, indicating continued gene regulation in these pathways. Two, eleven and seventeen additional pathways were specific to stage S3, S4 and S5, respectively (Fig. 3b, Supplementary Table S9). This suggested that longer SD treatment affected more pathways. The most enriched DE pathways included ribosome for translation, plant hormone signal transduction, carbon fixation in photosynthetic organisms for energy metabolism, and the circadian rhythm network. Change in ribosome for translation suggested altered protein synthesis. Here, we focused our subsequent analysis on the latter three pathways. No specific pathway was found in early stage S2 after only five days of SD treatment (Fig. 3b).

### Expression patterns of DE genes in hormone signal transduction

**Figure 4:**
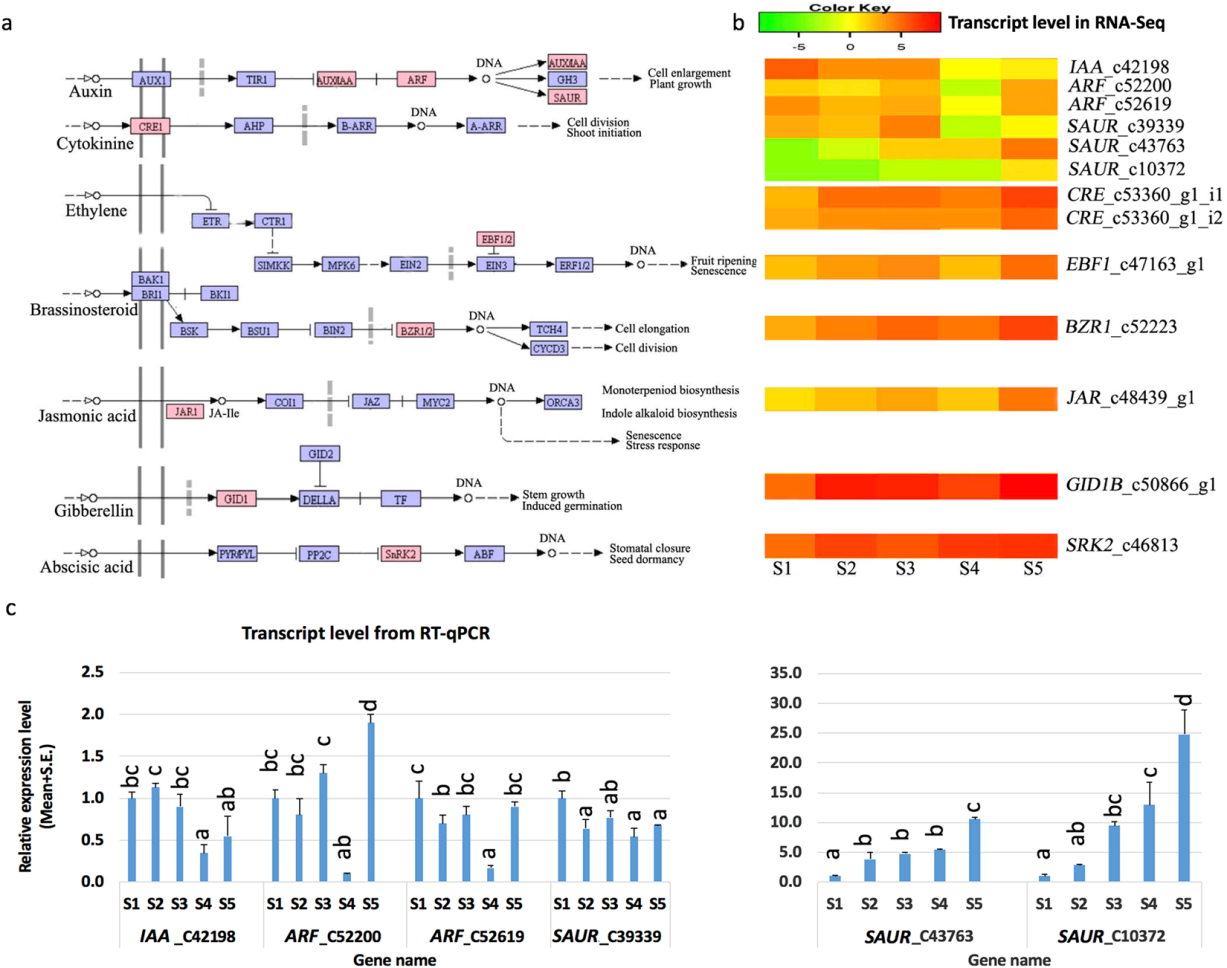
Regulation of genes in plant hormone signal transduction under SD. Image a shows the affected plant hormone network modified from map MAP04075 (version 9/6/16) in KEGG database (http://www.kegg.jp) by short-day photoperiods, and the shaded red box represented the differentially expressed genes. Image b shows the differential expression of involved genes in RNA-Seq analysis. Image c shows the transcript abundance measured with RT-qPCR. The Pearson coefficient between RNA-Seq result and RT-qPCR result is 0.9, 1, 0.9, 0.8, 1, 0.9 for *IAA_C42198*, *ARF*_*C52200*, *ARF_C52619*, *SAUR_C39339*, *SAUR_C43763*, *SAUR_C10372*, repectively. S1 represents the apical shoot tip samples or stage before treatment. S2, S3, S4, and S5 represent the apical shoot tip samples or stages after 5, 10, 15, and 20 days of short-day treatment.

We characterised the DEGs involved in plant hormone signal transduction. Further GO network enrichment analysis using Blast2GO (version 4.0.2) revealed that 12 DEGs were under the same GO accession (0009725), which is described as “in response to hormone” (Supplementary Fig. S2). KEGG pathway mapping with KAAS tool against *Arabidopsis* and *Populus* identified that these genes encode key proteins functioning in multiple hormone signalling pathways (Table 1), suggesting regulation of hormones under SD. The auxin pathway had the greatest number of enriched DEGs, and we annotated these gene functions based on protein homolog similarity (Table 1). Key genes encoding AUX/IAA, auxin response factor (ARF), and small auxin up RNA (SAUR) family proteins in the auxin pathway were regulated significantly in both RNA-Seq results and RT-qPCR results with good coefficients of 0.9, 1, 0.9, 0.8, 1, 0.9 for *IAA_C42198*, *ARF_C52200*, *ARF_C52619*, *SAUR_C39339*, *SAUR_C43763* and *SAUR_C10372*, respectively, suggesting a large impact on the auxin pathway (Fig. 4). The gene *IAA* was down-regulated through all stages with SD treatment and reached the lowest expression level at S4, after 15 days of treatment. Two copies of *ARF* were both down-regulated from S2 to S4 and then up-regulated at S5. As in other plants^37^, multiple *SAURs* were identified in the auxin pathway in *Paulownia* in this study. Two of three copies of *SAUR* were up-regulated, but at different rates, and the third copy displayed alternative up- and down-regulation (Fig. 4). In each of the other hormone pathways, only one DEG was regulated significantly during SD treatment, e.g. *CRE1* in the cytokinin pathway, *EBF1/2* in the auxin and ethylene pathway, *BZR1/2* in the brassinosteroid pathway, *JAR1* in the jasmonic acid pathway, *GID1* in the gibberellin pathway, and *SnRK2* in the abscisic acid pathway. The detailed gene expression patterns of these genes during each stage under SD were compared and are displayed in Fig. 4.

**Table 1:** Function of regulated genes in plant hormone signal transduction.

Hormone	Transcript^a^	KO ID	Function
Auxin	c42198_g1_i1	K14484	auxin-responsive protein (IAA)
	c52619_g1_i2	K14486	auxin response factor (ARF)
	c52200_g2_i3	K14486	auxin response factor (ARF)
	c10372_g1_i1	K14488	SAUR family protein (SAUR)
	c39339_g1_i1	K14488	SAUR family protein (SAUR)
	c43763_g1_i3	K14488	SAUR family protein (SAUR)
Cytokinin	c53360_g1_i2	K14489	cytokinine receptor (CRE)
	c53360_g1_i1	K14489	cytokinine receptor (CRE)
Abscisic acid	c46813_g3_i1	K14498	serine/threonine-protein kinase (SRK2)
Gibberellin	c50866_g1_i1	K14493	gibberellin receptor (GID1B)
Ethylene	c47163_g1_i1	K14515	EIN3-binding F-box protein 1 (EBF1)
Jasmonic acid	c48439_g1_i2	K14506	jasmonic acid-amino synthetase (JAR)
Brassinosteroid	c52223_g3_i1	K14503	brassinazole-resistant 1 protein (BZR1)

^a^The transcript is labeled by a suffix i# to a gene ID labeled by g#.

### Gene regulation in the circadian rhythmic network

**Figure 5:**
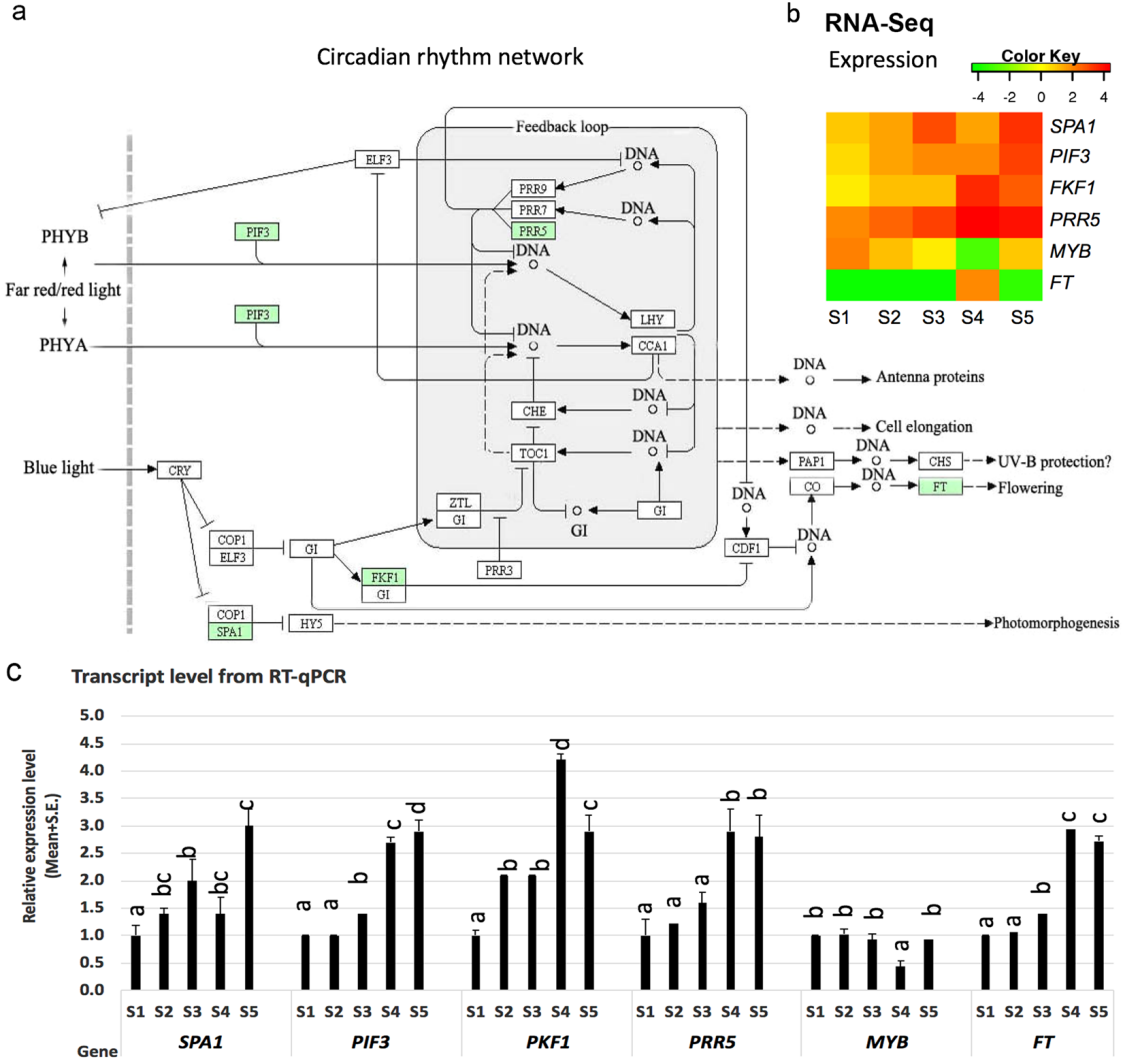
Differentially regulation of genes in circadian rhythm network in *Paulownia*. Image a shows the differentially expressed genes (shaded in small box) in circadian rhythm network, which are orthologs to *Arabidopsis* and *Populus*. The circadian rhythm network was modified from map MAP04712 (version 2012) from KEGG database (http://www.kegg.jp). Image b shows the expression level and pattern of genes from RNA-Seq data. Image c shows the relative expression patterns of genes measured with RT-qPCR. The Pearson coefficient between RNA-Seq result and RT-qPCR results is 1, 0.9, 1, 0.9, 0.9, 0.9 for gene *SPA1*, *PIF3*, *PKF1*, *PRR5*, *MYB*, *FT*, respectively.

Gene expression enrichment showed that many differentially expressed genes were in the circadian rhythm network. We examined the expression of these genes and mapped them to the well-documented circadian rhythm network of *Arabidopsis* and *Populus* with KAAS at the KEGG website. We identified and annotated six key genes, such as *PIF3*, *PRR5*, *PKF1*, *MYB* and *SAP1* (Table 2), with altered regulation in *Paulownia* under SD and mapped them onto the KEGG map MAP04712 (Fig. 5a). We validated the relative expression patterns with RT-qPCR which were consistent with RNA-Seq results with the coefficients of 1, 0.9, 1, 0.9, 0.9 and 0.9 for gene *SPA1*, *PIF3*, *PKF1*, *PRR5*, *MYB* and *FT*, respectively. Most of these genes showed up-regulated expression patterns but at different rates, except for *MYB* and *FT* (Fig. 5b). The expression of the key transcription factor *PIF3*, which functions in passing the SD signal from phytochromes to the internal clock loop, was up-regulated, which was consistent with previous reports in *Populus* under SD^3^ (Fig. 5a). *CO/FT* is responsible for seasonal dormancy in trees^3^, but we did not find significant changes in expression of *CO* under SD in one-year-old *Paulownia*. *FT* expression was very low under both LD and SD in RNA-Seq results, except that an up-regulated level of *FT* at a late stage, e.g. S4 (Fig. 5b,c), which was also reported previously in *Populus* under SD^3^.

**Table 2:** Function of regulated genes in circadian rhythm network.

Transcript^a^	KO ID	Function
c11143_g1_i1	K16223	protein FLOWERING LOCUS T (FT)
c38376_g1_i3	K16240	protein suppressor of PHYA-105 1 (SPA1)
c41412_g2_i1	K09422	Transcription factors (MYB)
c46583_g1_i2	K12116	flavin-binding kelch repeat F-box protein 1 (PKF1)
c50746_g1_i1	K12130	pseudo-response regulator 5 (PRR5)
c53430_g5_i1	K12126	phytochrome-interacting factor 3 (PIF3)

^a^The transcript is labeled by a suffix i# to a gene ID labeled by g#.

### Gene regulation in carbon fixation of photosynthesis under SD

**Figure 6:**
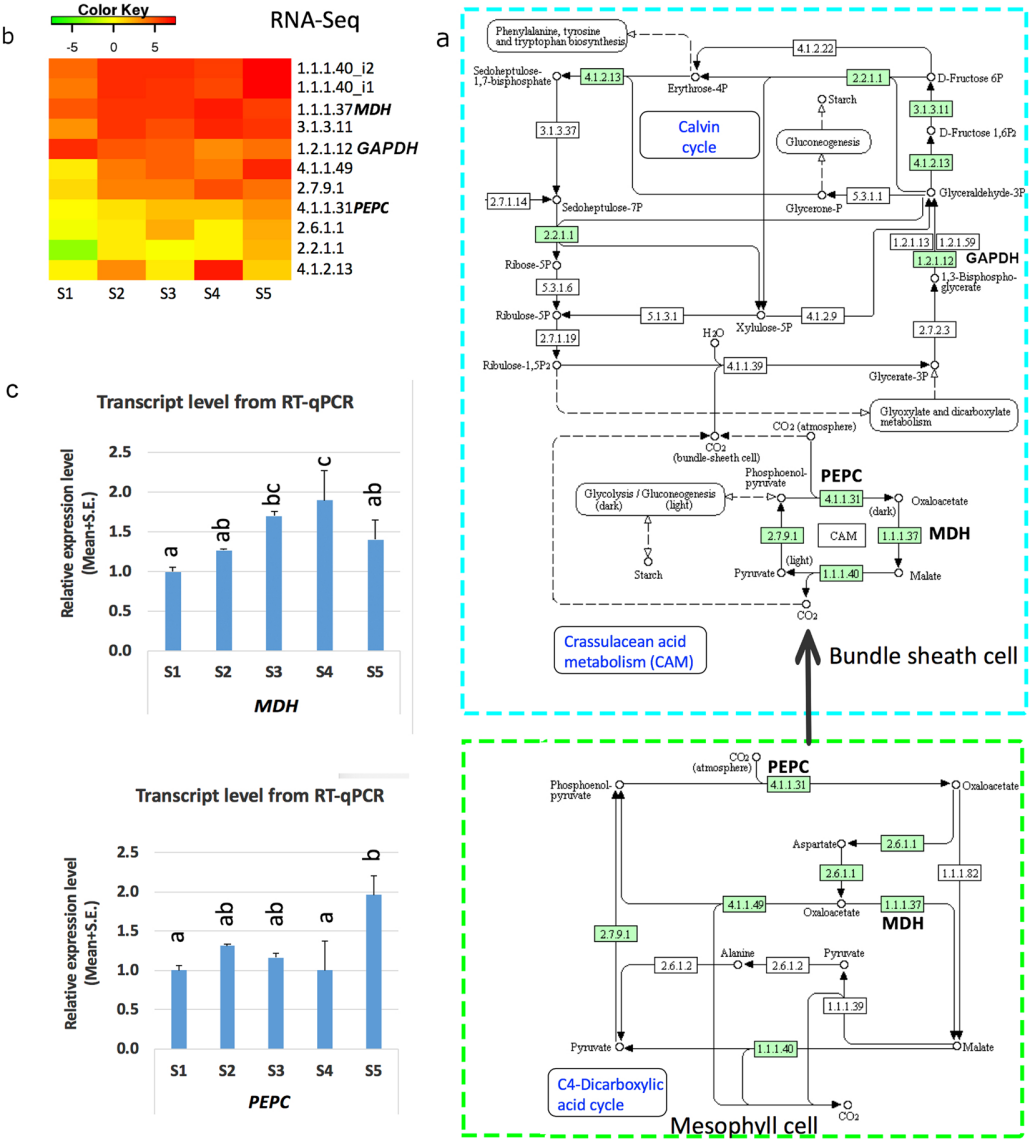
The differential gene regulation in carbon fixation of photosynthesis. Image a shows the metabolism of carbon fixation to which the differentially expressed genes (shaded) were mapped. The pathway was modified from map MAP00710 (version 2013) from KEGG database (http://www.kegg.jp). Image b shows the expression patterns of differential expressed genes encoding corresponding enzymes in pathway before (S1) and after (S2–S5) short day treatment. Image c shows the relative gene expression level detected with RT-qPCR. The pearson coefficient between RNA-Seq result and RT-qPCR results is 0.5 and 0.5 for *MDH* and *PEPC*, respectively.

**Figure 7:**
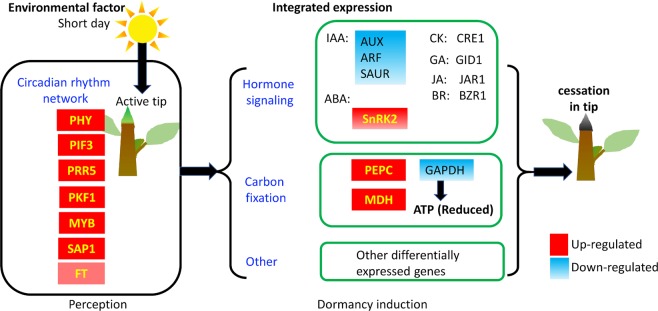
The schematic gene regulation incuded by short day treatment. Image shows the expressional regulation in response to short day treatment which induces growth cessation and dormancy in apical shoot tip.

To understand the role of DEGs in the enriched carbon fixation pathway, we further characterised these genes based on *Arabidopsis* and *Populus* in KEGG. Ten DEGs were mapped to three processes of the carbon fixation pathway of photosynthesis, including the C4-Dicarboxylic acid cycle, crassulacean acid metabolism (CAM), and the Calvin cycle (Fig. 6a). These DEGs encoded ten key enzymes catalysing the reactions of this pathway (Fig. 7, Supplementary Table S10). Most genes were upregulated, and the remaining genes showed dynamic up or down patterns (Fig. 6b). In the CO_2_ concentration process known in mesophyll cells, the gene *c53559_g1*, encoding PEPC EC:4.1.1.31 in the first step of binding CO_2_ to oxaloacetic acid, and the gene *c48076_g1* encoding malate dehydrogenase (MDH) EC:1.1.1.37 for the conversion of oxaloacetic acid to malate were up-regulated under SD. The expression patterns of *MDH* and *PEPC* in both RNA-Seq and RT-qPCR analysis showed a good correlation with coefficients of 0.5 and 0.5, respectively (Fig. 6b,c, Supplementary Table S10), suggesting a higher CO_2_-concentrating activity. All known genes encoding the enzymes in the known process CAM for CO_2_ residue delivery were upregulated. In the Calvin cycle, being the hub of metabolite conversion, the expression of six genes was altered. The gene *GAPDH* coding key enzyme glyceraldehyde 3-phosphate dehydrogenase (EC:1.2.1.12) was down-regulated, which indicated low glyceraldehyde-3-phosphate (G3P) generation from delivered CO_2_ (Fig. 6). Expression of the gene encoding Rubisco EC:4.1.1.39 was not changed.

## Discussion

Dormancy is an evolutionary adaptation to environmental factors, such as stresses, in perennial plants. It is known that an SD photoperiod can induce bud dormancy in some perennial plants^1^. Different from existing physiological reports on *Paulownia*^33^, here, we reported that SD, similar to winter days, induced growth cessation and dormancy, which mimics natural apical shoot tip in winter. Our transcriptome analyses identified DE genes and their regulation in pathways in *Paulownia* shoot tips under SD-induced growth cessation and dormancy induction in a time course during the fast-growing season. To our knowledge, this is a novel characterisation of the dynamics of global transcriptomes in response to SD treatment.

It had been thought that growth cessation, dormancy induction and bud formation are independent of each other^38,39^. Fifteen days of SD is sufficient to induce growth cessation and dormancy in the apical buds of *Paulownia*, and the buds could enter certain stages of dormancy. Previous report already shows that shoot tips stopped growth at the end of September under natural season and have enlarged vessel cells and increased xylem in the cambia zone of apical shoot^34^. The phenotype at stage S4 here was similar to that at the end of September. Therefore, the S4 stage should have growth cessation and could be at dormancy stage. The highest number of differentially expressed genes was approximately 20 days in our study under SD, which is comparable to the reported 21 days under SD in grapevine^5^. This indicated that 2–3 weeks may be enough to trigger most of the gene changes, which leads dormancy induction. A previous study showed that the leaf primordia in the apical meristem will grow into young leaves and then will die very soon, and the naked growth cone will gradually die too in the same cultivar during the dormancy processes^40^. Similar changes were observed in our study, which also supports SD-induced dormancy. The terminal shoot tips will die in the end, but we did not find any DE gene related to programmed cell death (PCD) in this study, which may be interpreted by our SD treatment at the fast-growing season or the induced cessation still at an early stage before PCD symptoms appeared.

Genetic and genomic studies on *Paulownia* lag far behind those on other plants, even though there have been several reports on gene expression relating to drought and hybridization of *Paulownia*^41,42^. Here, we enriched the transcripts and their gene expression in a time-course series, as well 491 commonly shared DE genes across all stages under SD, which will be the key set of gene regulated by SD induction although stage-specific genes may play roles too. The induced 116 shared pathways provide detailed regulation.

SD induces dormancy in perennial plants by down-regulating *CO* and *FT*, and up-regulating *PIF3/PIF4*^1^. The diurnal circadian rhythm plays crucial roles in the timing of gene expression to adapt to environmental conditions^43^. Our study found regulation of several circadian genes in SD-induced growth cessation and dormancy. The regulation occurred through genes in both the far red/red light and blue light signalling pathways and through interaction with circadian oscillators. These included significant changes (>2-fold) of five genes. *PIF3* was increasingly up-regulated in the far red and red signalling pathways. *SPA1* and *FKF1*, which control photomorphogenesis and interact with circadian oscillators in blue light, respectively, were regulated dynamically. PRR5 (pseudo-response regulator 5) is a transcriptional repressor for regulating the clock^44^, and its gene expression was up-regulated at all stages in the RNA-Seq analysis and, to our best knowledge, is the first observation of *PRR5* regulation under SD. The protein *MYB*, known to encode a circadian clock regulator^45^, was down-regulated through stages S2 to S4 and up-regulated at S5 after long SD treatment. We did not observe regulation of *CO* expression which was expected to be at low level at day time^1^ when the samples was collected, but we found that *FT* was regulated at late stage only. This may have been caused by the very young status of the one-year-old *Paulownia* plants we investigated here. A transcriptome analysis from SD to LD shows the *FT* is upregulated in *Medicago*^46^. Therefore, *FT* gene could be one of the key genes for dormancy induction under SD in apical shoot tips, which is of further interest in gene manipulation during breeding improvement.

Carbon fixation metabolism was also affected the growth cessation and dormancy induction in *Paulownia*. We found many DEGs involved in three processes of carbon fixation metabolism and genes in C4 model of carbon fixation in *Paulownia*. However, the Kranz anatomy of C4 plant was not present in *Paulownia*^47^. This is also reported that the Kranz anatomy is not essential in C4 plants^48^. The presence of expressed genes in both the C4 and CAM cycles suggest that *Paulownia* has evolved to separate initial CO2 fixation, minimize photorespiration and save water. The CAM is known to fix CO2 in the night which is beneficial to close stomata in the daytime to reduce water loss via transpiration^16,17^. Therefore, *Paulownia* may fix CO2 via CAM to adapt to hot and dry environments, which is good for the large leaf area of *Paulownia*. Leaf is the major tissue for photosynthesis while shoot tips should be minor tissue compared with leaves. However, the shoot tips of *Paulownia* are bigger than other plants and green, which may also conduct strong carbon fixation. In addition, many detected DEGs in photosynthesis suggested that the shoot tips do have photosynthesis and were affected. Whether these DEGs is the cause or the result of growth cessation is worthy of further investigation with the candidate genes identified here. Consistently, previous report shows that the carbon efflux was decreased at autumn in deciduous plants^19^. Thus, the photosynthesis is definitely associated with the growth cessation. During SD induction, the expression of the key gene *PEPC* was up-regulated, indicating an even higher efficiency of CO_2_ capture in the shoot tips. However, the gene *GAPDH*, which is responsible for converting released CO_2_ from malate to G3P, was down-regulated in the Calvin cycle. This suggested a high CO_2_ input but a low G3P output. Taking these data together, the extra captured carbon in the form of malate may be converted to another compound instead of going to the Calvin cycle under SD. The expression of *RUBISCO*, the most important gene in carbon fixation, was not changed in our study, although it was reported to be reduced in ABA-induced dormancy in *Spirodela*^21^. These may be explained by the fact that higher CO_2_-concentrating activity may suppress the decrease of *RUBISCO* expression in our study. Of course, posttranscriptional and translational modification will also affect the final activity of Rubisco.

Research efforts have investigated the role of hormones in inducing and maintaining dormancy and found that hormones often crosstalk with each other^7,8,12,15^. Microarray expression analysis showed genes in four hormones, including GA, ethylene, auxin and ABA, were regulated during bud dormancy in grape^9^. In our research, seven hormone pathways were significantly affected by SD treatment. The auxin signalling pathway showed the most significant changes because expression of six transcripts encoding *AUX/IAA*, *ARF* and *SAUR* genes was changed. Here, transcripts of one *IAA* and two copies of *ARF* were down-regulated and showed a dramatic downward trend at 15 days, which is consistent with those in previous transcriptomic studies during growth cessation and bud dormancy in other plants^6,7^, meaning common regulation in auxin pathways. *SAURs*, a gene family, are effectors that integrate environmental signals in the auxin pathway for plant growth by inhibiting ATPase and cell expansion, integrating other hormones, such as ABA, GA, Jasmonate, and Brassinosteroids (BR), to regulate plant growth and development^37^. In our results, three copies of *SAURs* displayed differential expression, meaning that they may have distinct regulation roles during plant development, as had been reported in a previous study^37^. Cytokinin synthesis was reported to be inhibited by auxin^9^. Two transcripts of *CRE* genes were differentially regulated. Taking these results together, we propose that the auxin signalling pathway has key roles in growth cessation and bud dormancy under SD in *Paulownia*.

Here our results show that genes in signalling of additional hormones, including BR, GA, JA, ABA and ethylene, were also regulated in growth cessation and bud dormancy. BR functions to promote stem elongation and cell division. Here, the up-regulated *BZR1* in SD treatment is a key transcription factor activated by BR. *BZR1* can interact with DELLA family proteins, which inhibit the GA signalling pathway^49,50^. Thus, BR signalling and GA signalling crosstalk under SD. JAR1, up-regulated in SD treatment, catalyses the synthesis of JA-amido conjugates, including the most active form, JA-Ile^51,52^. ABA is generally believed to play a positive role in seed and bud dormancy in woody plants^12^, and *SnRK2* is known to play an important role in ABA signalling^53^. Therefore, the up-regulated *SnRK2* in our study indicated an increase in ABA under SD to promote dormancy. Ethylene counteracts ABA signalling pathway and releases dormancy^15^. Here, the ethylene signalling pathway inhibitor encoding gene *EBF1* was up-regulated during SD treatment, which may be required for maintaining high ABA for the dormancy status. Together, many hormone-related pathway genes work together to induce and maintain growth cessation and dormancy under SD in *Paulownia*.

In summary, our RNA-Seq-based transcriptome analyses clarified that many genes were regulated in multiple pathways and metabolisms in apical shoot tips during SD-induced growth cessation and dormancy induction in *Paulownia*. The regulation of key genes in the circadian rhythm network, such as *PIF3* and *PRR5*, plays a role in environmental signal integration in dormancy induction. Expression of genes in all known plant hormones, especially *IAA*, *ARF* and *SAUR* in auxin signalling, was altered. Under SD, increased expression of *PEPC* was not coupled with higher assimilation to G3P in photosynthetic carbon fixation pathway. We summarized the discovery in this study in Fig. 7, where expressional regulation on key DE genes integrates the SD signal and leads to dormancy control in *Paulownia*. Our basic understanding of expressional regulation under SD in *Paulownia* provided here may help to guide dormancy management via editing key gene’s regulation for breeding higher quality timber.

## Materials and Methods

### Materials and treatment

To investigate short day effects on terminal growth of shoot tips, plant clones were generated from the *Paulownia* cultivar *P*. *tomentosa* roots buried in natural soil inside a greenhouse during the previous winter. The plants were grown until the beginning of Aug 2015, and the plants were chosen for this study when they were approximately 50–60 cm high with one healthy apical bud. Forty plants, eight plants for each sampling stage, were treated with short-day light cycles of 8 h light and 16 h darkness, while the natural day light cycle was 16 h light and 8 h darkness during the experiment. Approximately 0.5 cm of the shoot tip from each plant was sampled just before the treatment on Aug 1^st^ as a control, and then, the same tissues were sampled every five days (Aug 6, 11, 16 and 21). The samples were collected between 9 and 10 o’clock in the morning. The temperature was 27.8 °C, and the humidity was 65–85%. All plants were watered appropriately. The plants were grown in the greenhouse at Henan Agricultural University (GPS location 34.798 N, 113.650 E). Samples were then immediately frozen in liquid nitrogen and store at −80 °C until being processed.

### RNA extraction and sequencing

Shoot tips from the same sampling date were pooled and ground into powder with a mortar and pestle in liquid nitrogen. Total RNA was extracted from 80–120 mg powder for each sample using kit (TIANGEN, catalog # DP441) and checked in 1% agarose gel and then Agilent 2100 Bioanalyzer followed by subsequent Illumina library preparation as described previously^54^. Paired-end 125 bp sequencing was performed for each library on a HiSeq. 2500 machine.

### Transcript assembly, annotation and expression analysis

Before transcript assembly, raw reads were cleaned. The removal of poor-quality sequences and trimming of adaptor sequences were carried out using cutadapt (v1.8.1). The clean reads were merged and assembled into transcripts using Trinity software (version 201308)^55^ with the default parameters except with the parameters–min_kmer_cov 3–min_glue 3. All transcripts were then clustered by cdhit (v4.6) with default parameters^56^. Then, the clustered transcripts were aligned to the Rfam database (v.11; http://www.sanger.ac.uk/Software/Rfm/) to exclude reads corresponding to rRNAs. The software package RSEM was adopted to quantify total expressed transcripts in FPKM (fragments per kilobase of exon model per million mapped reads)^57^. The read count was used for differentially expressed genes analysis with EdgeR package (version 3.14) with settings as at least two-fold change and qvalue < 0.0001^58^. The expression patterns of genes across sampling stages were clustered with mfuzz package (version 2.44)^59^. Unigenes were defined as the longest sequences in the assembly cluster called component in Trinity^55^. Unigenes were then searched against the databases of NR, KEGG and EggNOG with BLAST with E-value threshold 1E-5. Gene function analysis was predicted using InterProScan software (version 5.8–49)^60^, and enrichment analysis was done using WEGO^61^ and Blast2GO software with default settings^62^. KEGG mapping and enrichment were analysed using KOBAS with default settings^36^ and annotated with the Automatic Annotation Server at the KEGG website (www.genome.jp/kegg/) using default settings against *Arabidopsis* and *Populus* species^63,64^. The permission to publish KEGG pathway map images of Circadian rhythm, Carbon fixation in photosynthetic organisms and Plant hormone signal transduction is granted by Kanehisa Laboratories.

### Real-time quantitative (RT-qPCR) analysis

RT-qPCR analysis was conducted for selected DEGs to validate the expressional patterns mined from RNA-Seq analysis. Total RNAs were extracted from shoot tips of triplicate biological plants with a RNAprep Pure kit (TIANGEN, catalog # DP441) and cDNA was synthesized from mRNA with FastQuant RT kit (TIANGEN, catalog # KR106) as described previously^54^. Three independent experiments were conducted. Primers are designed from DEGs with primer3 and provided in supplementary file (Supplementary Table S11). The primers efficiency in RT-qPCR was optimized and efficiency was tested in dilution series of 1, 1/10, 1/1000 and 1/10000 (Supplementary Table S11, Supplementary Fig. S3). RT-qPCR analysis was conducted for selected DEGs using SYBR green master according to previous methods except annealing temperature set to 60 °C here^65^. 18 S and H_2_O was used as reference gene and blank controls. The relative expression level was calculated with 2-ΔΔCt method^66^. The relative expression levels were presented as mean value ± standard error (S.E.) relative to S1. Therefore, the RT-qPCR results show the trend of expression pattern, but not the absolute level. Different letters indicate statistically significant differences to Duncan’s multiple range tests with *p* < 0.05. The coefficient between RNA-Seq result and RT-qPCR result was calculated with Pearson correlation.

## Supplementary information

Supplementary Information

Table S6

Table S5

Table S8

Table S4

Table S7

## Data Availability

The RNA-Seq reads and transcriptome assembly have also been deposited at the Short Read Archive and DDBJ/ENA/GenBank under the Bioproject accessions PRJNA393208 and GFVK00000000, respectively. The version described in this paper is the first version, GFVK01000000.
