# Supplementary material for: Time-coursed transcriptome analysis identifies key expressional regulation in growth cessation and dormancy induced by short days in Paulownia
Source: Sci Rep. 2019 Nov 12;9:16602. doi: 10.1038/s41598-019-53283-2 (PMC6851391; doi:10.1038/s41598-019-53283-2)
Supplement: Supplementary file 1 — Supplementary Information [file 41598_2019_53283_MOESM1_ESM.pdf]

Supplementary information

# Time-coursed transcriptome analysis identifies key expressional regulation in growth cessation and dormancy induced by short days in *Paulownia*

Jiayuan Wang[1], Hongyan Wang[2], Tao Deng[3], Zhen Liu[1*], Xuewen Wang[3,4]*

Table of Contents

SUPPLEMENTARY INFORMATION 1

SUPPLEMENTAL TABLES 2

SUPPLEMENTARY TABLE S1 SUMMARY OF RNA-SEQ READS, ASSEMBLY AND ANNOTATION IN *PAULOWNIA* 2
SUPPLEMENTARY TABLE S2 EGGNOG FUNCTIONAL ANNOTATION FOR UNIGENES 3
SUPPLEMENTARY TABLE S3 THE NUMBER OF DE GENES UNDER SHORT-DAY TREATMENT 4
SUPPLEMENTARY TABLE S4 ALL DEGS ID AND UP OR DOWNREGULATION 5
SUPPLEMENTARY TABLE S5 DEG ANNOTATION RESULTS FROM DATABASE NR 5
SUPPLEMENTARY TABLE S6 DEG ANNOTATION RESULTS FROM DATABASE INTERPRO AND GO 5
SUPPLEMENTARY TABLE S7 DEG ANNOTATION RESULTS FROM DATABASE EGGNOG 5
SUPPLEMENTARY TABLE S8 DEG ANNOTATION RESULTS FROM DATABASE KEGG 5
SUPPLEMENTARY TABLE S9 THE LIST OF 209 ENRICHED KEGG PATHWAYS 5
SUPPLEMENTARY TABLE S10 FUNCTIONS OF MAPPED GENES INTO CARBON FIXATION PATHWAY 10
SUPPLEMENTARY TABLE S11 PRIMERS AND AMPLIFICATION EFFICIENCY IN RT-QPCR ANALYSIS 11

SUPPLEMENTAL FIGURES 12

*SUPPLEMENTARY FIGURE S1* COMPARISON OF GENE EXPRESSION PROFILE BETWEEN STAGES UNDER SD 12
SUPPLEMENTARY FIGURE S2 GO NETWORK ENRICHMENT ANALYSIS FOR ALL HORMONE DE GENES 13
SUPPLEMENTARY FIGURE S3 RT-QPCR AMPLIFICATION STANDARD CURVE 13

Supplemental Tables

**Supplementary Table S1** Summary of RNA-seq reads, assembly and annotation in *Paulownia*

| | Category | Data | | | | |
|---|---|---|---|---|---|---|
| RNA-seq reads | Sample ID | S1 | S2 | S3 | S4 | S5 |
| | SAR accession | SRR5813993 | SRR5813994 | SRR5813995 | SRR5813996 | SRR5813997 |
| | Reads | 43,793,744 | 42,639,826 | 41,819,430 | 39,734,274 | 51,632,694 |
| | GC(%) | 44 | 44 | 45 | 45 | 44 |
| | Q30(%) | 93.41 | 93.63 | 93.04 | 93.38 | 93.51 |
| | Total reads | 219,619,968 | | | | |
| Assembly | | | | | | |
| | Total genes | | | | 49,054 | |
| | Total transcripts | | | | 67,879 | |
| | Total length (bp) | | | 72,610,969 | | |
| | Mean length (bp) | | | | 1,068 | |
| | Sequence count (length 201-400 bp) | | | | 24,356 | (35.9%) |
| | Sequence count (length 1000 bp) | | | | 27,316 | (40.2%) |
| Annotation | | | | | | |
| | Total annotated transcripts | | | | 54,880 | (80.8%) |
| | NR (%) | | | | 30,653 | (45.1%) |
| | EggNOG (%) | | | | 24,184 | (35.6%) |
| | KEGG (%) | | | | 6,647 | (9.8%) |
| | InterPro (%) | | | | 24,378 | (35.9%) |

S1 represent the stage of long day (16 hr light and 8 darkness) just before the short-day treatment. S2, S3, S4, and S5 represent the samples after 5, 10, 15, and 20 days of short-day treatment (8 hr light and 16 darkness). Data are public available at NCBI with a master accession PRJNA393208.

**Supplementary Table S2 EggNog functional annotation for unigenes**

| #eggNOG_class | subclass | Numbers | total |
|---|---|---|---|
| INFORMATION STORAGE AND PROCESSING | Translation, ribosomal structure and biogenesis | 2290 | |
| INFORMATION STORAGE AND PROCESSING | Transcription | 3540 | |
| INFORMATION STORAGE AND PROCESSING | Chromatin structure and dynamics | 582 | |
| INFORMATION STORAGE AND PROCESSING | RNA processing and modification | 947 | |
| INFORMATION STORAGE AND PROCESSING | Replication, recombination and repair | 1791 | |
| **INFORMATION STORAGE AND PROCESSING Total** | | **9150** | **28%** |
| METABOLISM | Coenzyme transport and metabolism | 589 | |
| METABOLISM | Lipid transport and metabolism | 1403 | |
| METABOLISM | Energy production and conversion | 1263 | |
| METABOLISM | Inorganic ion transport and metabolism | 1060 | |
| METABOLISM | Secondary metabolites biosynthesis, transport and catabolism | 890 | |
| METABOLISM | Carbohydrate transport and metabolism | 2191 | |
| METABOLISM | Amino acid transport and metabolism | 1568 | |
| METABOLISM | Nucleotide transport and metabolism | 511 | |
| **METABOLISM Total** | | **9475** | **29%** |
| CELLULAR PROCESSES AND SIGNALING | Signal transduction mechanisms | 3167 | |
| CELLULAR PROCESSES AND SIGNALING | Defense mechanisms | 396 | |
| CELLULAR PROCESSES AND SIGNALING | Posttranslational modification, protein turnover, chaperones | 4812 | |
| CELLULAR PROCESSES AND SIGNALING | Nuclear structure | 5 | |

| | | | |
|---|---|---|---|
| CELLULAR PROCESSES AND SIGNALING | Cell cycle control, cell division, chromosome partitioning | 764 | |
| CELLULAR PROCESSES AND SIGNALING | Cell wall/membrane/envelope biogenesis | 773 | |
| CELLULAR PROCESSES AND SIGNALING | Cytoskeleton | 1073 | |
| CELLULAR PROCESSES AND SIGNALING | Cell motility | 30 | |
| CELLULAR PROCESSES AND SIGNALING | Intracellular trafficking, secretion, and vesicular transport | 3155 | |
| **CELLULAR PROCESSES AND SIGNALING Total** | | **14175** | **43%** |
| **Grand Total** | | **32800** | |

**Supplementary Table S3 The number of DE genes under short-day treatment**

| Levels | Stages | Up_regulated genes | Down_regulated genes | Sub-total |
|---|---|---|---|---|
| Fold of change: >2 | | | | |
| P <0.0001 | S2 | 330 | 349 | 679 |
| | S3 | 615 | 437 | 1052 |
| | S4 | 592 | 625 | 1217 |
| | S5 | 699 | 579 | 1278 |
| | Total unique | 1540 | | |
| Fold of change: >4 | | | | |
| P <0.0001 | S2 | 330 | 349 | 679 |
| | S3 | 615 | 437 | 1052 |
| | S4 | 592 | 625 | 1217 |
| | S5 | 699 | 579 | 1278 |
| | Total unique | 1540 | | |
| Fold of change: >8 | | | | |
| P <0.0001 | S2 | 293 | 342 | 635 |
| | S3 | 544 | 412 | 956 |
| | S4 | 518 | 547 | 1065 |
| | S5 | 617 | 529 | 1146 |
| | Total unique | 1346 | | |

**Supplementary Table S4 All DEGs id and up or downregulation**

File: TableS4_all.DEG.updown.log2P0.0001.xlsx

**Supplementary Table S5 DEG annotation results from database NR**

File: TableS5_DEG.Nr.anno.xls

**Supplementary Table S6 DEG annotation results from database Interpro and GO**

File: TableS6_DEG.interproGO.anno.csv

**Supplementary Table S7 DEG annotation results from database EggNog**

File: TableS7_eggNOG.ann.xlsx

**Supplementary Table S8 DEG annotation results from database KEGG**

File: TableS8_DEG.Kegg.anno.xls

**Supplementary Table S9 The list of 209 enriched KEGG pathways**

| Stages | Sub_total | KEGG names |
|---|---|---|
| S2 S3 S4 S5 | 116 | Inositol phosphate metabolism |
| | | Pantothenate and CoA biosynthesis |
| | | Regulation of autophagy |
| | | NOD-like receptor signaling pathway |
| | | Cutin, suberine and wax biosynthesis |
| | | 2-Oxocarboxylic acid metabolism |
| | | Phototransduction |
| | | Biosynthesis of unsaturated fatty acids |
| | | Pyruvate metabolism |
| | | Citrate cycle (TCA cycle) |
| | | FoxO signaling pathway |
| | | Pyrimidine metabolism |
| | | Peroxisome |
| | | Fatty acid degradation |
| | | Starch and sucrose metabolism |
| | | Metabolism of xenobiotics by cytochrome P450 |
| | | Drug metabolism - cytochrome P450 |
| | | Sulfur metabolism |
| | | PPAR signaling pathway |
| | | Olfactory transduction |
| | | Tyrosine metabolism |
| | | mTOR signaling pathway |
| | | Rap1 signaling pathway |
| | | Vascular smooth muscle contraction |
| | | alpha-Linolenic acid metabolism |

Tropane, piperidine and pyridine alkaloid biosynthesis
Ubiquitin mediated proteolysis
Dopaminergic synapse
Degradation of aromatic compounds
Gastric acid secretion
beta-Alanine metabolism
MAPK signaling pathway
Cysteine and methionine metabolism
Pentose phosphate pathway
cGMP - PKG signaling pathway
Proximal tubule bicarbonate reclamation
PI3K-Akt signaling pathway
Oxytocin signaling pathway
Salivary secretion
Circadian rhythm
Arginine and proline metabolism
Alanine, aspartate and glutamate metabolism
mRNA surveillance pathway
Collecting duct acid secretion
Plant hormone signal transduction
Two-component system
Carotenoid biosynthesis
D-Glutamine and D-glutamate metabolism
Ras signaling pathway
Melanogenesis
cAMP signaling pathway
Protein processing in endoplasmic reticulum
Gap junction
Valine, leucine and isoleucine biosynthesis
Phenylpropanoid biosynthesis
RNA transport
Phosphatidylinositol signaling system
p53 signaling pathway
GnRH signaling pathway
Inflammatory mediator regulation of TRP channels
Cyanoamino acid metabolism
HIF-1 signaling pathway
Estrogen signaling pathway
Phagosome
Adipocytokine signaling pathway

Apoptosis
Oocyte meiosis
Carbon metabolism
Carbon fixation in photosynthetic organisms
Neurotrophin signaling pathway
Monoterpenoid biosynthesis
Glyoxylate and dicarboxylate metabolism
Selenocompound metabolism
Insulin signaling pathway
Isoquinoline alkaloid biosynthesis
Progesterone-mediated oocyte maturation
Methane metabolism
Retinol metabolism
Biosynthesis of amino acids
Zeatin biosynthesis
Renin secretion
Flavonoid biosynthesis
Circadian entrainment
Flavone and flavonol biosynthesis
RNA degradation
Galactose metabolism
Diterpenoid biosynthesis
Arginine biosynthesis
Antigen processing and presentation
Lysine degradation
Ribosome
Carbon fixation pathways in prokaryotes
Synaptic vesicle cycle
Fatty acid metabolism
Long-term potentiation
Phototransduction - fly
Chloroalkane and chloroalkene degradation
Vitamin B6 metabolism
AMPK signaling pathway
Fructose and mannose metabolism
Oxidative phosphorylation
Nitrogen metabolism
Glycolysis / Gluconeogenesis
Phenylalanine metabolism
Adrenergic signaling in cardiomyocytes

| | | |
|---|---|---|
| | | Glucagon signaling pathway |
| | | Purine metabolism |
| | | Spliceosome |
| | | Endocytosis |
| | | Glutathione metabolism |
| | | Valine, leucine and isoleucine degradation |
| | | Naphthalene degradation |
| | | Sesquiterpenoid and triterpenoid biosynthesis |
| | | Plant-pathogen interaction |
| | | Glycine, serine and threonine metabolism |
| | | Calcium signaling pathway |
| S2 S3 S4 | 1 | Streptomycin biosynthesis |
| S2 S4 S5 | 5 | Biosynthesis of ansamycins |
| | | Nicotinate and nicotinamide metabolism |
| | | Ribosome biogenesis in eukaryotes |
| | | Nucleotide excision repair |
| | | Brassinosteroid biosynthesis |
| S3 S4 S5 | 26 | Glycerolipid metabolism |
| | | Histidine metabolism |
| | | Glucosinolate biosynthesis |
| | | Tryptophan metabolism |
| | | Thiamine metabolism |
| | | Stilbenoid, diarylheptanoid and gingerol biosynthesis |
| | | Phenylalanine, tyrosine and tryptophan biosynthesis |
| | | Taurine and hypotaurine metabolism |
| | | Ascorbate and aldarate metabolism |
| | | Butanoate metabolism |
| | | Endocrine and other factor-regulated calcium reabsorption |
| | | Isoflavonoid biosynthesis |
| | | ABC transporters |
| | | GABAergic synapse |
| | | Steroid biosynthesis |
| | | Terpenoid backbone biosynthesis |
| | | Porphyrin and chlorophyll metabolism |
| | | Bile secretion |
| | | Amino sugar and nucleotide sugar metabolism |
| | | Limonene and pinene degradation |
| | | Lysosome |
| | | Pentose and glucuronate interconversions |

| | | |
|---|---|---|
| | | Ubiquinone and other terpenoid-quinone biosynthesis |
| | | Tetracycline biosynthesis |
| | | Propanoate metabolism |
| | | Fatty acid biosynthesis |
| S2 S4 | 4 | DNA replication |
| | | Mismatch repair |
| | | Base excision repair |
| | | Homologous recombination |
| S3 S4 | 1 | Thyroid hormone synthesis |
| S3 S5 | 7 | Glycosphingolipid biosynthesis - globo series |
| | | One carbon pool by folate |
| | | Other glycan degradation |
| | | Novobiocin biosynthesis |
| | | Photosynthesis |
| | | Sphingolipid metabolism |
| | | Linoleic acid metabolism |
| S4 S5 | 19 | Drug metabolism - other enzymes |
| | | Glycerophospholipid metabolism |
| | | Cell cycle - yeast |
| | | Circadian rhythm - plant |
| | | Polyketide sugar unit biosynthesis |
| | | Cell cycle |
| | | Biotin metabolism |
| | | Ether lipid metabolism |
| | | Meiosis - yeast |
| | | Wnt signaling pathway |
| | | Arachidonic acid metabolism |
| | | Aminobenzoate degradation |
| | | Fc gamma R-mediated phagocytosis |
| | | Anthocyanin biosynthesis |
| | | Pancreatic secretion |
| | | SNARE interactions in vesicular transport |
| | | TGF-beta signaling pathway |
| | | Sphingolipid signaling pathway |
| | | MAPK signaling pathway - yeast |
| S3 | 2 | Carbohydrate digestion and absorption |
| | | Butirosin and neomycin biosynthesis |
| S4 | 11 | Vasopressin-regulated water reabsorption |
| | | Non-homologous end-joining |
| | | Glutamatergic synapse |

| | | |
|---|---|---|
| | | Bisphenol degradation |
| | | Riboflavin metabolism |
| | | Glycosaminoglycan degradation |
| | | Photosynthesis - antenna proteins |
| | | Protein export |
| | | Polycyclic aromatic hydrocarbon degradation |
| | | Fatty acid elongation |
| | | Fanconi anemia pathway |
| S5 | 17 | Axon guidance |
| | | Chemokine signaling pathway |
| | | Toll-like receptor signaling pathway |
| | | Osteoclast differentiation |
| | | Cardiac muscle contraction |
| | | Regulation of actin cytoskeleton |
| | | Caffeine metabolism |
| | | RNA polymerase |
| | | C5-Branched dibasic acid metabolism |
| | | Styrene degradation |
| | | Focal adhesion |
| | | Leukocyte transendothelial migration |
| | | Adherens junction |
| | | B cell receptor signaling pathway |
| | | Fc epsilon RI signaling pathway |
| | | VEGF signaling pathway |
| | | Natural killer cell mediated cytotoxicity |
| total | 209 | |

**Supplementary Table S10 Functions of mapped genes into carbon fixation pathway**

| Gene_Transcript(g#_i#) | KO ID | Function |
|---|---|---|
| c24652_g1_i1 | K00615 | transketolase [EC:2.2.1.1] |
| c26634_g1_i1 | K01623 | fructose-bisphosphate aldolase, class I [EC:4.1.2.13] |
| c38227_g1_i1 | K14454 | aspartate aminotransferase, cytoplasmic [EC:2.6.1.1] |
| c42914_g1_i1 | K01006 | pyruvate, orthophosphate dikinase [EC:2.7.9.1] |
| c44467_g1_i1 | K03841 | fructose-1,6-bisphosphatase I [EC:3.1.3.11] |
| c48076_g1_i2 | K00026 | malate dehydrogenase [EC:1.1.1.37], MDH |
| c52094_g1_i1 | K00029 | malate dehydrogenase (oxaloacetate-decarboxylating)(NADP+) [EC:1.1.1.40 |
| c52094_g1_i2 | K00029 | malate dehydrogenase (oxaloacetate-decarboxylating) (NADP+) [EC:1.1.1.40] |

| | | |
|---|---|---|
| c52785_g1_i1 | K00134 | glyceraldehyde 3-phosphate dehydrogenase [EC:1.2.1.12], GAPDH |
| c53096_g2_i1 | K01610 | phosphoenolpyruvate carboxykinase (ATP) [EC:4.1.1.49] |
| c53559_g1_i5 | K01595 | phosphoenolpyruvate carboxylase [EC:4.1.1.31], PEPC |

g# for gene ID, _i# for transcript ID

## Supplementary Table S11 Primers and amplification efficiency in RT-qPCR analysis

| Gene name | ID | Forward (5'-3') primer | Reverse (5'-3') primer | Efficiency (%) |
|---|---|---|---|---|
| *18S* | Pau-18s | ACATAGTAAGGATTGACAGA | TAACGGAATTAACCAGACA | 87.4 |
| *MDH* | c48076_g1_i2 | CCCAACCACAGGAACATTGACTT | GGAATTGCAAAGTGCTGTCCTAA | 110.3 |
| *PEPC* | c53559_g1_i5 | GGTTTTTTACATGGCACTCCCGT | CGTCCACTGTTTGGACCTGACCT | 91.1 |
| *IAA* | c42198_g1_i1 | TTCTGATTTTTGTGACGGTGAGG | CAATGCCAAAACAGCCAAAGAG | 109.5 |
| *ARF* | c52619_g1_i2 | CAGAGACTTTGCTTCTTGCGGTA | GGAGCACAAACAGAATGGGAAA | 110.9 |
| *ARF* | c52200_g2_i3 | AACTATCCAAATCTCCCCCCTCA | GCCGTCAAAGTTTTGCAGAAGAA | 109.9 |
| *SAUR* | c10372_g1_i1 | ATGTGGTTGTCGGCTGAATCCTA | CATTCCAGTAGCAGTTGGCGTG | 80.6 |
| *SAUR* | c39339_g1_i1 | CATTTTGCTGTGATTGCTGAGGA | TTATCAACACCACCACCAGCAAC | 96.6 |
| *SAUR* | c43763_g1_i3 | AATCACCATCATTTTGACCCACA | GCTTAACCCACCTCAAATCATCC | 83.2 |
| *FT* | c11143_g1_i1 | ACGGGGGAACCAAGATTGTAGAG | CAGCAACCACTGGAGCAACTTTT | 121.5 |
| *SPA1* | c38376_g1_i3 | GAGCATAGTGAAAGGGCTTGGTC | AGCTAAAAGCCAACAAATGAGAGG | 109.9 |
| *MYB* | c41412_g2_i1 | CAACATCATAAGACCCCGACCTC | GCTGCTTGTTCTTGGGATTCTCA | 117.0 |
| *PKF1* | c46583_g1_i2 | AACACTCACCCTACGCCACTCTG | AATGACTAAAAAGTTGGGATGGGG | 88.7 |
| *PRR5* | c50746_g1_i1 | GGTCTATGTTACGGGGTCCTCCT | CTGTTGTGAGGTGGGAGAGGTTT | 92.5 |
| *PIF3* | c53430_g5_i1 | TCAACCGTTCAATGAGCAAGGAT | GGAGAGGCAGAATATAACGCTGC | 80.5 |

Note: annealing Tm=60 °C.

## Supplemental Figures

*Supplementary Figure S1* Comparison of gene expression profile between stages under SD

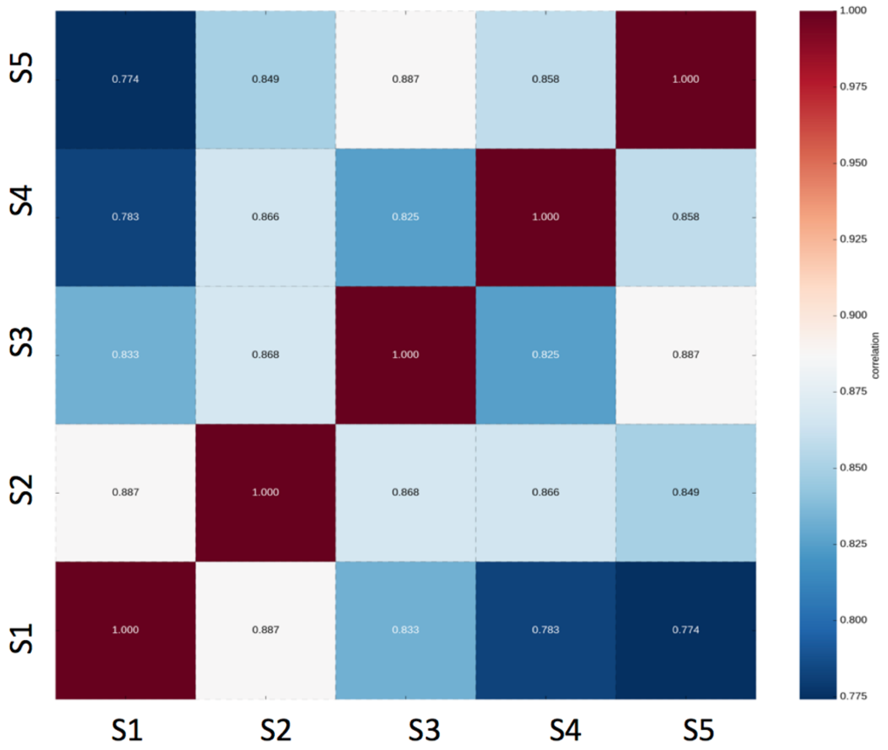


Images shows the correlation of the number of expressed genes. X axis represent stages the five-day interval while the plants were treated of short-day-light (8 hr light and 16 hr darkness) cycles. Stages 1 (S1) was the control at the starting point at long day cycles of 16 hr light and 8 hr darkness. Label S2, S3, S4, and S5 represent the samples after 5, 10, 15, and 20 days of short-day treatment.

Supplementary Figure S2 GO network enrichment analysis for all hormone DE genes

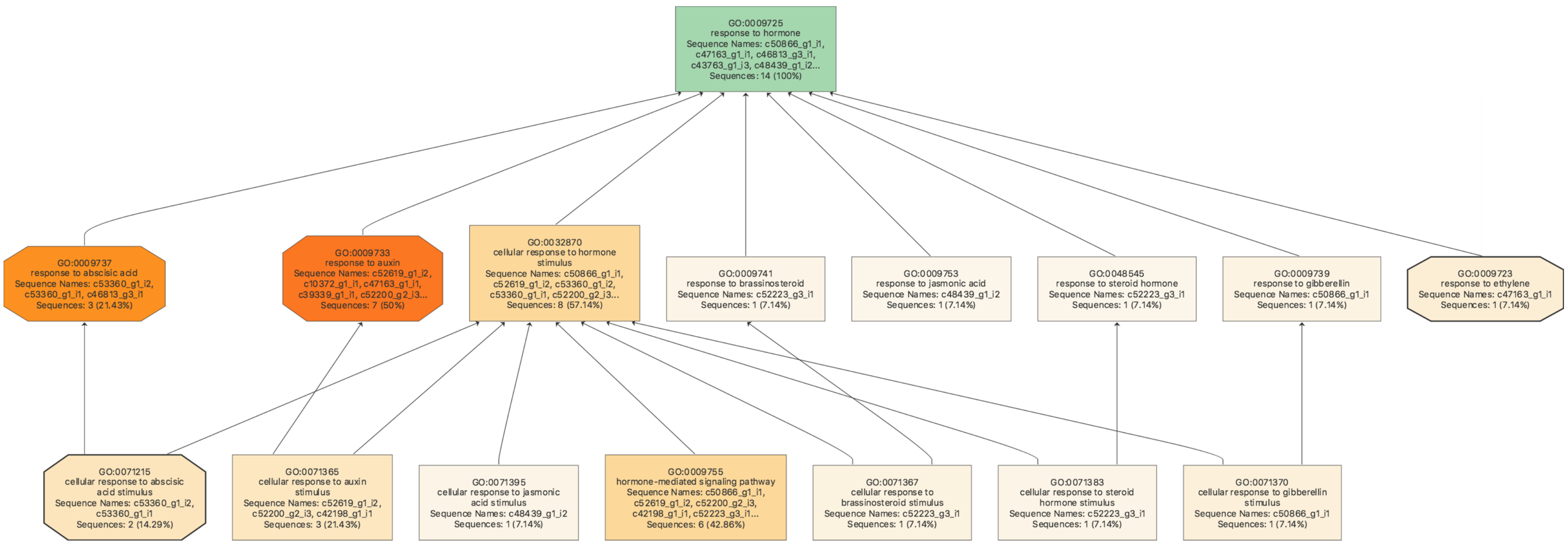


Supplementary Figure S3 RT-qPCR amplification standard curve

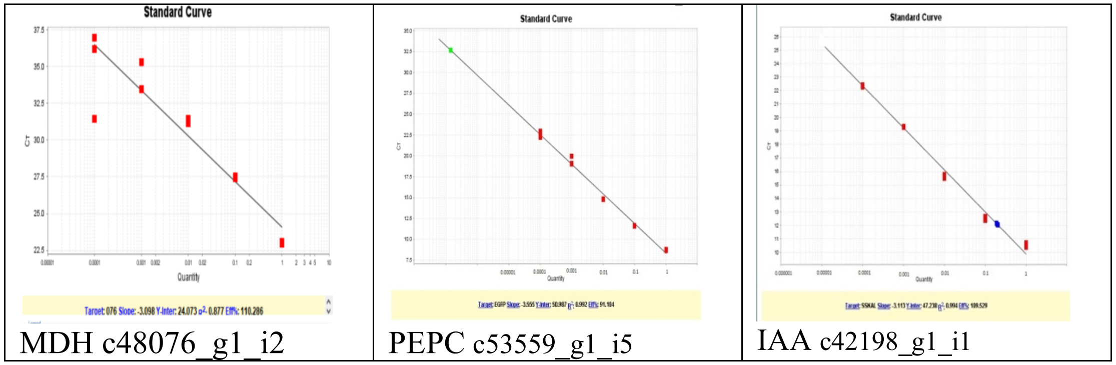

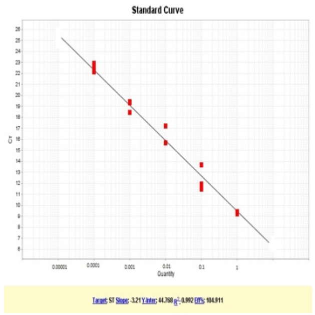


ARF c52619_g1_i2

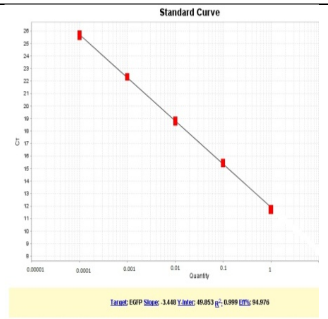


ARF c52200_g2_i3

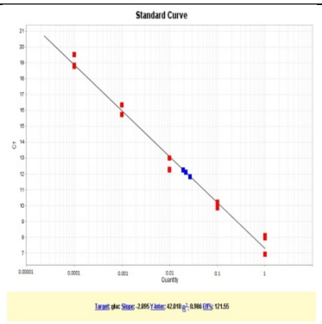


SAUR c10372_g1_i1

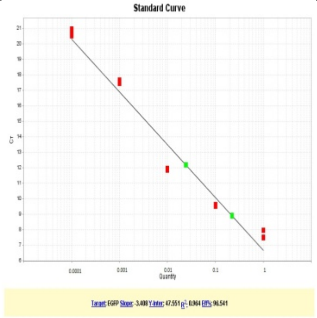


SAUR c39339_g1_i1

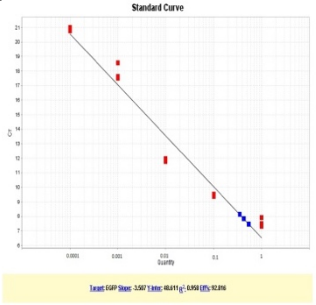


SAUR c43763_g1_i3

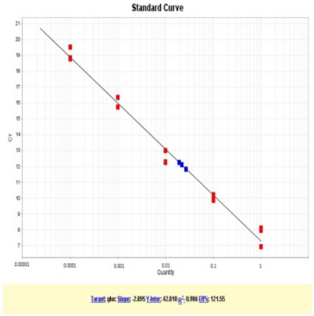


FT c11143_g1_i1

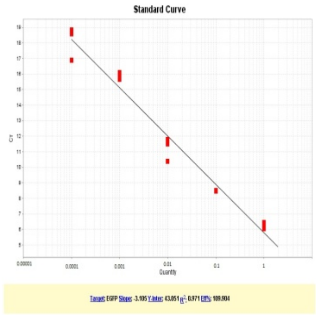


SPA1 c38376_g1_i3

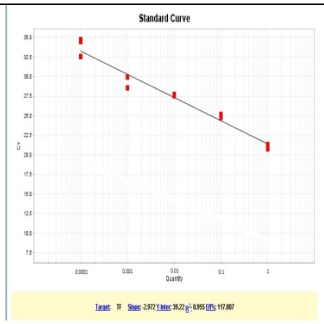


MYB c41412_g2_i1

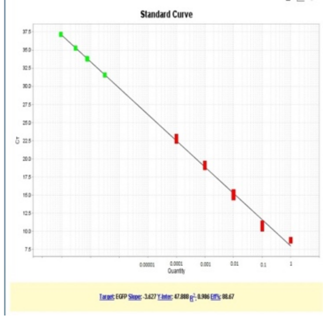


PKF1 c46583_g1_i2

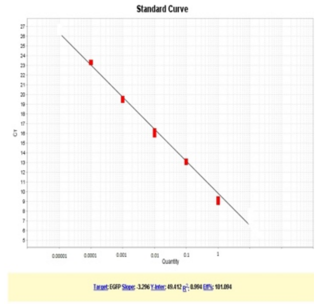


PRR5 c50746_g1_i1

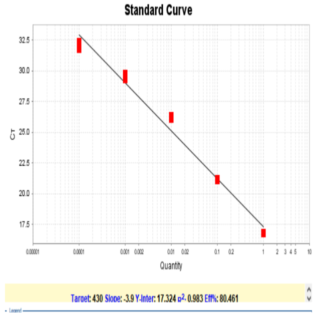


PIF3 c53430_g5_i1

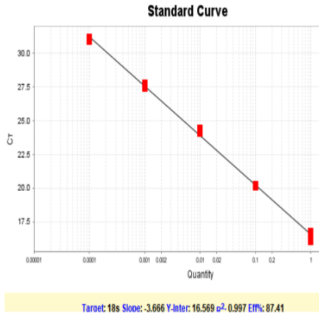


Pau-18s

The cDNA was diluted into series of 1, 1/10, 1/1000 and 1/10000. Three replicates were conducted with RT-qPCR amplification for each diluted cDNA for each primer pair. The amplification curve was plotted with mean Ct values and amplification efficiency was calculated by the formula $(10^{(-1/slope)}-1) * 100\%$ . The name below each curve represent the gene name and primer ID.
